# A high-resolution radiation hybrid map of chicken chromosome 5 and comparison with human chromosomes

**DOI:** 10.1186/1471-2164-5-66

**Published:** 2004-09-15

**Authors:** Frédérique Pitel, Behnam Abasht, Mireille Morisson, Richard PMA Crooijmans, Florence Vignoles, Sophie Leroux, Katia Feve, Suzanne Bardes, Denis Milan, Sandrine Lagarrigue, Martien AM Groenen, Madeleine Douaire, Alain Vignal

**Affiliations:** 1 Laboratoire de Génétique Cellulaire, INRA, Castanet-Tolosan, 31326, France; 2 UMR Génétique Animale, INRA-ENSAR, Route de St Brieuc, Rennes, 35042, France; 3 Animal Breeding and Genetics group, Wageningen University, Wageningen, 6709 PG, The Netherlands

## Abstract

**Background:**

The resolution of radiation hybrid (RH) maps is intermediate between that of the genetic and BAC (Bacterial Artificial Chromosome) contig maps. Moreover, once framework RH maps of a genome have been constructed, a quick location of markers by simple PCR on the RH panel is possible. The chicken ChickRH6 panel recently produced was used here to construct a high resolution RH map of chicken GGA5. To confirm the validity of the map and to provide valuable comparative mapping information, both markers from the genetic map and a high number of ESTs (Expressed Sequence Tags) were used. Finally, this RH map was used for testing the accuracy of the chicken genome assembly for chromosome 5.

**Results:**

A total of 169 markers (21 microsatellites and 148 ESTs) were typed on the ChickRH6 RH panel, of which 134 were assigned to GGA5. The final map is composed of 73 framework markers extending over a 1315.6 cR distance. The remaining 61 markers were placed alongside the framework markers within confidence intervals.

**Conclusion:**

The high resolution framework map obtained in this study has markers covering the entire chicken chromosome 5 and reveals the existence of a high number of rearrangements when compared to the human genome. Only two discrepancies were observed in relation to the sequence assembly recently reported for this chromosome.

## Background

Chicken is the first major agricultural species for which the complete genome sequencing was undertaken. This is partly due to its position as a model species in various fields of biology including embryo development, oncology, immunology and evolution [1]. Moreover, as it is the only bird species for which the genome study is so advanced, very much is expected from its use in comparative genome analyses for annotation, including that of the human genome, by detection of conserved sequences [2,3]. Its intermediate phylogenetic position between mammals and fishes will also certainly provide valuable information on the evolution of vertebrate karyotypes.

Radiation hybrid maps have a resolution power intermediate to that of the genetic and BAC contig maps and are also a powerful tool for the mapping of ESTs and genes by simple PCR. They are thus useful at two levels: first, they can be used constructively as scaffolds for a correct genome assembly or for detecting and correcting misassembled portions of the genome; second, before obtaining whole annotated genome sequences, they are very efficient tools for inter-species comparative genome analyses through the easy mapping of genes and ESTs [4-7].

The successful production of a RH panel in chicken is quite recent [8], and therefore RH maps are only available for a limited number of chromosomes [9-11]. Having identified QTL (Quantitative Trait Loci) for fatness on chicken chromosome 5 [12], our objective was to build a high-resolution and gene-rich RH map for this chromosome, as a basis for high precision comparative mapping with human and for the development of new polymorphic markers.

The available human/chicken comparative mapping data indicated conservation of synteny between GGA5 and portions of HSA11, HSA14 and HSA15. In addition, two genes from HSA1 had also been shown to be located on GGA5 [13]. This information was used to develop markers from chicken EST sequence data orthologous to genes in these human regions, in addition to the existing markers from the chicken chromosome 5 genetic map.

While in the process of finishing our map, the first draft sequence assembly of the chicken genome was released (March 1^st^, 2004). The quality of both the GGA5 RH map and of the sequence assembly was therefore checked by alignment of all the markers by BLAST searches.

## Results and discussion

### Development of EST markers

In addition to the 21 microsatellite markers from the genetic map, and 9 primer pairs chosen either from available primer data in the literature or designed using the gene sequence deposited in Genbank/EMBL, 156 primer pairs were chosen from chicken EST markers selected on the basis of the known conservations of synteny between human and chicken using the ICCARE (Interactive Comparative Clustering and Annotation foR Est) software . Constraints on the design of primers were to avoid presence of long introns, whose position and length was predicted on the basis of the orthologous human gene structure, and to design primers in the most divergent regions of the human/chicken alignment, to limit cross-amplification with the hamster DNA present in the hybrids. One hundred and thirty nine primer pairs out of 156 (89.1%) enabled a successful amplification and the subsequent mapping of the corresponding genes, confirming the high success rate obtained when using the ICCARE software for designing chicken PCR primers based on EST data [10].

### Construction of the GGA5 RH map

**Figure 1:**
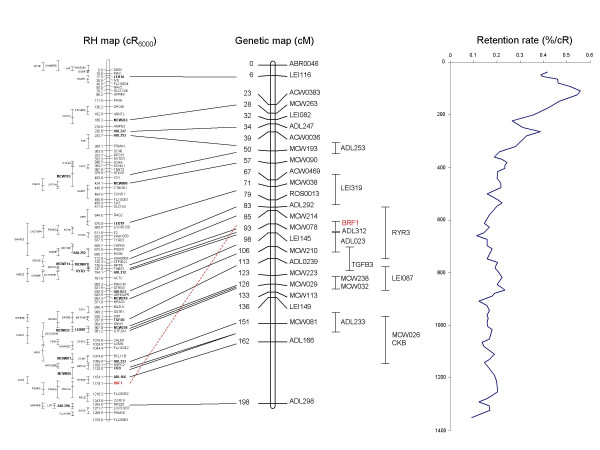
**Comparison RH / genetic maps for chicken chromosome 5. **The framework RH map is 1315.6 cR_6000 _long. Position of markers included only in the comprehensive map is indicated with error bars to the left of the framework map. Markers for which the genetic position is known (Schmid et al, 2000) are indicated by links to the genetic map (middle). Retention frequency along the map is represented on the right.

Altogether, genotyping data was obtained for a total of 169 markers, comprising 148 gene fragments (of which 139 developed using ICCARE) and 21 microsatellites from the GGA5 genetic map. Two-point analysis using a LOD threshold of 6 enabled to constitute a group of 134 markers, including all the microsatellite markers from the genetic map. The remaining 35 markers correspond to the external boundaries of the regions of conserved synteny with human, from which ESTs were chosen for marker development and map either to other chromosomes for which RH maps were developed (GGA1, 10, 18 or 24) or to unknown regions (data not shown). After multipoint analysis, a 1000:1 framework map 1315.6 cR_6000 _long, comprising a total of 73 markers including 12 microsatellites and 61 ESTs was obtained. The remaining 61 markers are located relative to the framework map within confidence intervals, to build a comprehensive map (figure 1).

To compare the RH and the genetic maps, the best possible position of the non-framework common markers had to be estimated. That of the markers on the RH map was computed by the Carthagene program and is indicated in addition to the confidence interval. For the genetic map, the central position of the marker's confidence interval was used as their most probable position. As a result, the order of the markers on the RH map matches exactly that of the same markers on genetic map [13], with only one notable discrepancy concerning the position of *BRF1 *(figure 1). However, when the position of this gene was checked on the sequence assembly, the agreement was with the RH map, suggesting the position of this gene on the genetic map is erroneous.

An average retention frequency of 21.4% was observed for the 134 GGA5 markers studied here, although with a high variation, with values ranging from 6.8% to 55.7%. This finding is within the range observed in other studies reported on this panel: 21.9% overall retention using 42 markers chosen genome-wise [8], 24 % for GGA4 [11], 20.1 for GGA7 [10] and 18% for GGA15 [9]. As already noticed for several species including human [14,15] or cow [16], but also for chicken chromosomes 4 and 7 [10,11], a centromeric effect is detected when observing retention frequencies of markers along the map, with a higher retention of markers in the region between 50 to 200 cR, in which the retention culminates at a value of 55.7%, whereas it is around 15% for the rest of the chromosome (400 cR downwards).

### Alignment of the RH map to the genomic sequence

**Figure 2:**
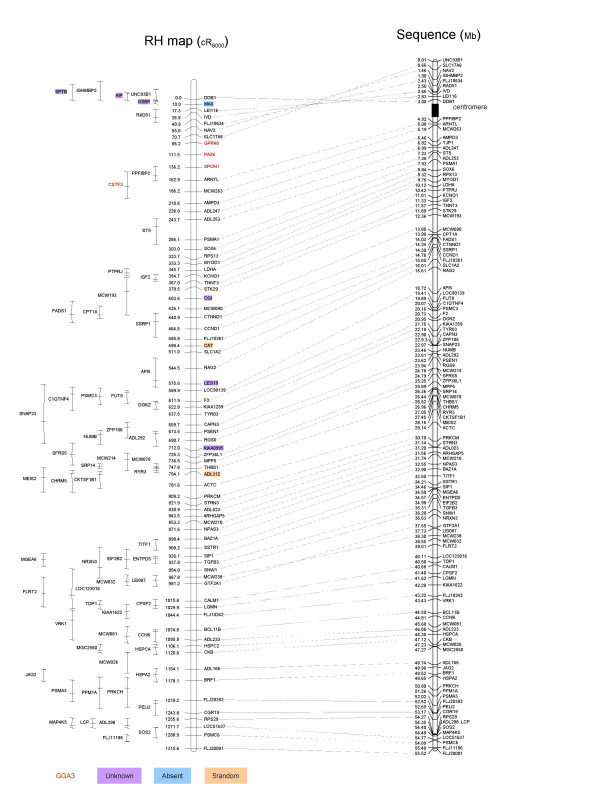
**Comparison between RH map and chicken genome assembly. **The RH map (left) obtained in this study is compared to the draft sequence assembly (right, ). For each marker on the framework map, a line joins both positions (cR and Mb) together. Discrepancies or missing data are indicated. Unknown: sequence of unknown location in the assembly; absent: sequence not found (no BLAST hit); 5_random: sequence attributed to GGA5, but whose position is unknown precisely in the assembly.

A preliminary data set based on the first draft chicken genome assembly has been deposited into public databases by a team led by R. Wilson and W. Warren, from the Washington University School of Medicine in St. Louis (1st March, 2004, ). We compared our data with the GGA5 sequence, by using BLASTN searches and sequence alignments. The agreement between the RH framework map and the sequence orders is almost perfect (figure 2), although with a few discrepancies, most of them suggesting possible improvements to be made in the sequence assembly.

First, a group of markers (*GPR48*, *PAX6*, *SPON1 *and *CSTF3*), that we developed on the basis of the conservation of synteny between GGA5 and HSA11, is assigned to GGA3 in the sequence assembly. Three of these markers are on the framework map and for all four, the RH genotypings obtained are very similar to those obtained with the flanking RH framework markers *SLC17A6 *and *ARNTL *(two-point LOD scores ranging from 7.1 to 15.7), both located on the GGA5 sequence assembly. Furthermore, when two-point analysis of the four markers was computed with the flanking markers *LOC134957 *(1.2 Mb to *GPR48*) and *SLC22A3 *(0.6 Mb to *CSTF3*) suggested in the GGA3 sequence assembly, LOD scores were equal to zero. This part of the genome assigned to a wrong chromosome on the sequence assembly covers a region at least 50 cR long, corresponding to a distance of 2 to 3 Mb, as estimated from the cR to Mb ratio. Indeed, the length of the sequence between the two extreme markers *CSTF3 *and *GPR48 *on the GGA3 assembly is 2.703 Mb. The retention frequency of these four markers is amongst the highest of all, suggesting that their location is close to the centromere and that the possible sequence assembly problems are related to this proximity, perhaps due to repetitive sequences.

Second, we observed an inversion of the gene order for a segment in the upper part of the chromosome (first 86.2 cR or 3.08 Mb, figure 2) adjacent to the group we described as wrongly assigned to GGA3 in the sequence assembly. As the difference of likelihood between our 1000:1 framework map and the map order in this area suggested from the assembly is higher than 10^15^, we considered that order of the RH map is the correct one. This could also be due to assembly difficulties close to the centromere region.

Third, several markers absent in the sequence assembly could be localised on the RH map (figure 2). Most of these markers belong to regions for which sequence information is available, but that couldn't be incorporated in the sequence assembly at all (Unknown) or that could be assigned to GGA5, but without a clear location (5_random). In addition, one gene (*MAX*) also appeared to belong to a region with no sequence available (no blast hit). This gene had previously been located on the cytogenetic map to the short arm of GGA5 [17], so we consider our data as a confirmation.

Fourth, we observed a discrepancy in the local order of the two framework map markers MCW238 and *GTF2A1*. However, the difference of likelihood between our framework map and the same map with an inversion of these two markers is only 10^3.7^. It is therefore difficult to conclude as to which between the sequence and the RH map presents the correct order.

From these data we conclude that radiation hybrid maps can be useful to help detect errors in the draft sequence assembly and for mapping genes either absent or of unknown location in the assembly.

### Comparison cR_6000_/cM/kb

The average cR/cM ratio is 6.5 when calculated over the whole map length. This relatively high value, as compared to the 4 cR/cM obtained for GGA7 [10], must be inflected by the disparity observed along the chromosome (figure 1). This heterogeneity actually reflects disparities in the recombination rate along the chromosome, with recombination events more frequent at the end of the long arm.

The agreement between the gene order found on RH map and the sequence assembly is very high. Considering only the q arm of the chromosome, the cR/Mb ratio is 22.9, or 43.7 kb per cR. This ratio, similar to that obtained for GGA2 (S. Leroux, personal communication), is quite lower than the 63 kb/cR and 61 kb/cR values obtained for GGA15 [9] and GGA7 [10] respectively, suggesting a higher resolution for the larger chromosomes. This result can have two origins: first, the kb/cR ratio is not constant from one chromosome to another, regardless of their physical length [18,19,14]; second, the previous calculations were based on physical length values estimated from cytogenetic studies: 21 Mb for GGA15 [20] and 41 Mb for GGA7 [21]. If we consider the actual chromosome length based on sequence assembly, these chromosomes are shorter than previously estimated, with values of 12.4 and 37.3 Mb , the ratio is thus now closer to the value we obtain here for GGA5.

### Comparative mapping

**Figure 3:**
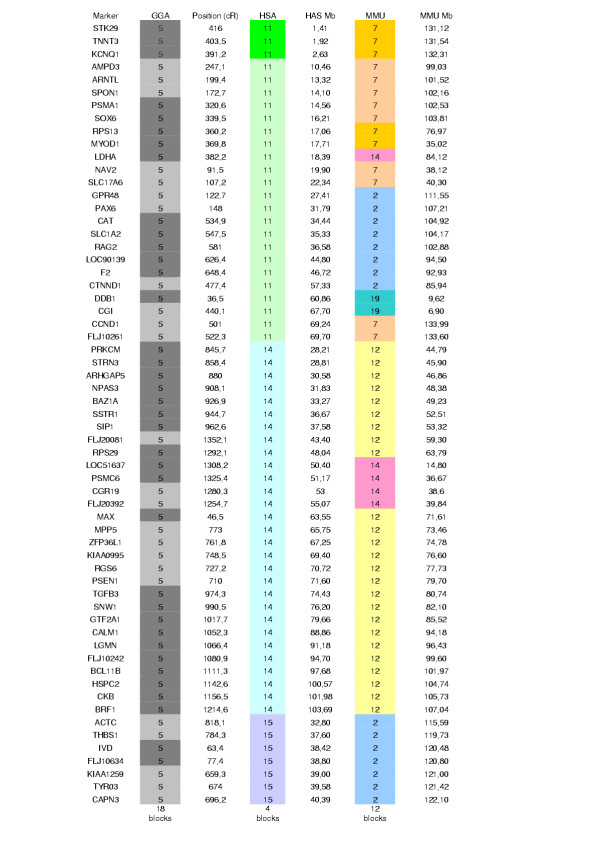
**Comparative positions between chicken, human and mouse genomes for the framework map genes. **The position of each gene on the chicken, human and mouse maps is given: chicken chromosome (GGA), cR position (this study); human chromosome (HSA), Mb position, and mouse chromosome (MMU), Mb position. The position used for human and mouse genes are from EnsMart v19.1 (human build 34, update v19.34a.1; mouse build 30, update v19.30.1 – ). Coloured blocks indicate the blocks of conserved gene order, using the human as reference.

Figure 3 and figure 4 (see additional file 2) synthesize the comparative maps generated by us between GGA5 and its human and mouse counterparts. As indicated earlier [13,22-28], conserved synteny was observed between this chicken chromosome and portions of human chromosomes 11, 14 and 15. No correspondence was detected with HSA1, as is also supported by the GGA5 sequence assembly . The results indicate a high number of chromosomal rearrangements in the chicken and human lineages in the region corresponding to GGA5. The results presented in figures 3 and 4 make us conclude that, as previously observed [10,29], the number of synteny blocks is higher between chicken and mouse than between chicken and human. The high number of intra-chromosomal rearrangements within the regions of conserved synteny between birds and mammals is in accordance with results obtained for other chromosomes, e.g., GGA7 [10], GGA10 [25], GGA15 [20], and chicken regions homologous to HSA19 [30].

## Conclusions

We have built a high resolution RH map of chicken chromosome 5 using the ChickRH6 panel. In doing this, we fulfilled our objective of obtaining a detailed comparative map of GGA5, providing jointly a source of potential polymorphic markers and of candidate genes for QTL mapping on this chromosome.

At the end of our work, the first draft chicken genome assembly was released and we aligned it to our GGA5 RH map. Although we detected a few errors to correct, this allowed us to demonstrate the high quality of the sequence assembly, which may have benefited from a low frequency of repeated elements.

In the near future, the ChickRH6 panel will be used to assist in improving the chicken genome assembly. This is clearly needed in the regions for which the genetic map is still not complete, such as some microchromosomes, but also for parts of macrochromosomes, as shown in this study.

## Methods

### Development of markers

Twenty one microsatellite markers distributed along GGA5 were chosen from the genetic map. Their primer sequences are available at .

Human and mouse genes from regions for which available comparative mapping data suggested a conservation of synteny with GGA5 were selected for marker development. Except for *CKB*, *IGF2 *and *RYR3 *for which primers were chosen from the literature, and 6 other genes for which primers were designed from sequences deposited in Genbank/EMBL, primers pairs were designed from the available chicken EST sequence of orthologs defined using the ICCARE (Interspecific Comparative Clustering and Annotation foR ESTs) software (T. Faraut, ). The exonic structure of the genes was taken into account by extrapolating the information available from an alignment to the human genomic sequence. A link with the Primer3 software  allowed us to design the primers. Primer data for markers amplifying successfully and accession numbers of the sequences used as a basis for primer design, are indicated in Table 1 (see additional file 1).

### Radiation hybrids – PCR amplification

The generation of the RH panel has already been described [8]. The final panel is composed of 90 clones with an average retention frequency of 21.9%.

PCR amplifications were carried out for each marker in 15 μl reactions containing 25 ng DNA, 0.2 μM of each primer, 0.3 U of Taq polymerase (Life Technologies-GIBCO BRL), 20 mM Tris-HCl pH 8.4, 50 mM KCl, 0.05% W-1 detergent, 2 mM MgCl2, 0.2 mM dNTP.

Amplifications were carried out on a GeneAmp PCR System 9700 thermocycler (Applied Biosystem). The first 5 min denaturation was followed by 30 cycles, each of denaturation at 94°C for 30 s, annealing at Tm for 30 s and elongation at 72°C for 30 s. PCR products were analyzed on 2% agarose gels, electrophoresed in 1 X TBE buffer, and visualized by ethidium bromide staining.

Each marker was genotyped twice and a third genotyping was performed in cases of discrepancies between the first two experiments.

### Map construction

The genotyping data obtained was analyzed with the Carthagene software [31,32]. A group of GGA5 markers was defined by two-point analysis using a LOD threshold of 6. By using all the markers from this group, a 1000:1 framework map (a map whose likelihood is at least 1000 fold higher than the next possible highest likelihood using the same markers in alternate orders) was built under a haploid model. This framework was constructed using a stepwise locus adding strategy, starting from the triplet of markers whose order is the most likely ("buildfw" option). The framework map thus automatically built was further improved towards larger distance coverage by removing markers that prevented its extension. The different provisional framework maps were checked by using a simulated annealing greedy algorithm testing for possible improvements of the map by inversion of large fragments, and a flips algorithm testing all possible local permutations within a sliding window of six markers. After validation of the framework map built under the haploid model, the distances between markers of the framework were re-evaluated under a diploid model. Finally, markers not included in the framework map were mapped relative to it, to determine their most likely positions.

The human and mouse reference maps were built from data available through EnsMart v19.1 (14^th ^January 2004 – ). RH maps were drawn with MapChart 2.0 [33].

### Sequence comparison

Sequences for all the mapped fragments were used for a BLAST search over the entire chicken genome assembly at the Ensembl chicken site  to determine their position in the sequence. The sequence assembly map of our markers was visualised with MapChart 2.0 [33].

## Authors' contributions

FP and BA carried out most of the molecular studies. FP drafted the manuscript. MM made the RH panel. RC and MG were involved in the GGA5 study. FV, SL, KF and SB were involved in the characterization of the panel. We use the Carthagene software thanks to DM. Construction of the maps was done after fruitful discussions with MM and SL. AV and MD conceived the study, and participated in its design and coordination. AV finalised the manuscript. All authors read and approved the final manuscript.

## Supplementary Material

Additional File 2 **Comparative maps of chicken chromosome 5 and human chromosomes 11, 14 and 15. **The framework RH map (this study) is shown on the left. Conserved blocks are indicated by coloured plain boxes. Empty boxes show HSA regions for which the chicken homologous part of the genome is not positioned on GGA5. Click here for file

Additional File 1 **Primer pairs for the studied gene fragments **Accession numbers for the chicken EST sequences from which the primers were chosen are given in this Excel file Click here for file
