# Supplementary material for: A high-resolution radiation hybrid map of chicken chromosome 5 and comparison with human chromosomes
Source: BMC Genomics. 2004 Sep 15;5:66. doi: 10.1186/1471-2164-5-66 (PMC521070; doi:10.1186/1471-2164-5-66)
Supplement: Additional File 2 — Comparative maps of chicken chromosome 5 and human chromosomes 11, 14 and 15. The framework RH map (this study) is shown on the left. Conserved blocks are indicated by coloured plain boxes. Empty boxes show HSA regions for which the chicken homologous part of the genome is not positioned on GGA5. [file 1471-2164-5-66-S2.ppt]

## Slide 1
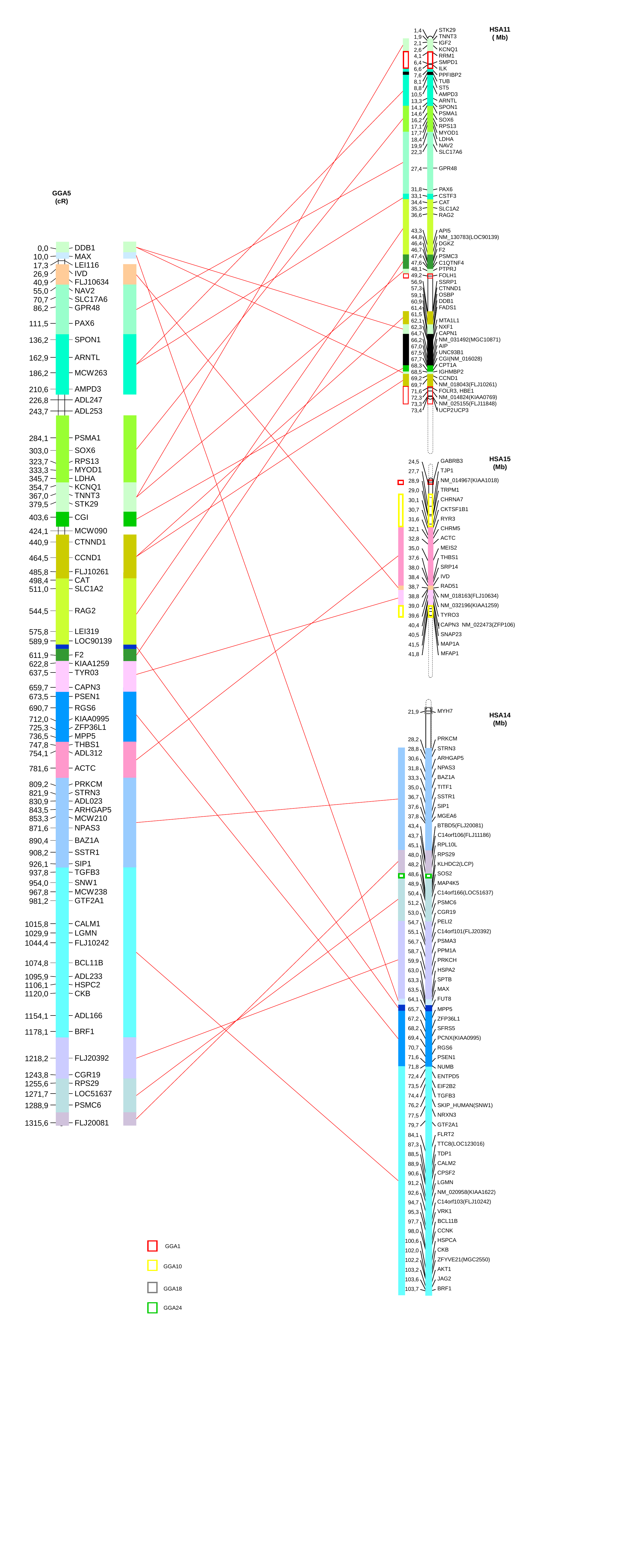

HSA11
( Mb)
STK29
1,4
TNNT3
1,9
IGF2
2,1
KCNQ1
2,6
RRM1
4,1
SMPD1
6,4
ILK
6,6
PPFIBP2
7,6
TUB
8,1
ST5
8,8
AMPD3
10,5
ARNTL
13,3
SPON1
14,1
PSMA1
14,6
SOX6
16,2
RPS13
17,1
MYOD1
17,7
LDHA
18,4
NAV2
19,9
SLC17A6
22,3
GPR48
27,4
31,8
PAX6
GGA5
(cR)
33,1
CSTF3
34,4
CAT
35,3
SLC1A2
36,6
RAG2
43,3
API5
44,8
NM_130783(LOC90139)
46,4
DGKZ
46,7
F2
47,4
PSMC3
47,6
C1QTNF4
48,1
PTPRJ
49,2
FOLH1
56,9
SSRP1
57,3
CTNND1
OSBP
59,1
DDB1
60,9
FADS1
61,4
61,5
MTA1L1
62,1
NXF1
62,3
CAPN1
64,7
NM_031492(MGC10871)
66,2
AIP
67,0
UNC93B1
67,5
CGI(NM_016028)
67,7
CPT1A
68,3
IGHMBP2
68,5
CCND1
69,2
NM_018043(FLJ10261)
69,7
FOLR3, HBE1
71,6
NM_014824(KIAA0769)
72,3
NM_025155(FLJ11848)
73,3
UCP2
UCP3
73,4
HSA15
(Mb)
GABRB3
24,5
TJP1
27,7
NM_014967(KIAA1018)
28,9
TRPM1
29,0
CHRNA7
30,1
CKTSF1B1
30,7
RYR3
31,6
CHRM5
32,1
ACTC
32,8
MEIS2
35,0
THBS1
37,6
SRP14
38,0
IVD
38,4
RAD51
38,7
NM_018163(FLJ10634)
38,8
NM_032196(KIAA1259)
39,0
TYRO3
39,6
CAPN3
NM_022473(ZFP106)
40,4
SNAP23
40,5
MAP1A
41,5
MFAP1
41,8
MYH7
21,9
HSA14
(Mb)
PRKCM
28,2
STRN3
28,8
ARHGAP5
30,6
NPAS3
31,8
BAZ1A
33,3
TITF1
35,0
SSTR1
36,7
SIP1
37,6
MGEA6
37,8
BTBD5(FLJ20081)
43,4
C14orf106(FLJ11186)
43,7
RPL10L
45,1
RPS29
48,0
KLHDC2(LCP)
48,2
SOS2
48,6
MAP4K5
48,9
C14orf166(LOC51637)
50,4
PSMC6
51,2
CGR19
53,0
PELI2
54,7
C14orf101(FLJ20392)
55,1
PSMA3
56,7
PPM1A
58,7
PRKCH
59,9
HSPA2
63,0
SPTB
63,3
MAX
63,5
FUT8
64,1
65,7
MPP5
67,2
ZFP36L1
68,2
SFRS5
69,4
PCNX(KIAA0995)
70,7
RGS6
71,6
PSEN1
71,8
NUMB
72,4
ENTPD5
73,5
EIF2B2
74,4
TGFB3
76,2
SKIP_HUMAN(SNW1)
NRXN3
77,5
GTF2A1
79,7
FLRT2
84,1
TTC8(LOC123016)
87,3
TDP1
88,5
CALM2
88,9
CPSF2
90,6
LGMN
91,2
NM_020958(KIAA1622)
92,6
C14orf103(FLJ10242)
94,7
VRK1
95,3
BCL11B
97,7
CCNK
98,0
HSPCA
100,6
GGA1
CKB
102,0
ZFYVE21(MGC2550)
102,2
GGA10
AKT1
103,2
JAG2
103,6
GGA18
BRF1
103,7
GGA24
